# Estimating the risk of irreversible post-SSRI sexual dysfunction (PSSD) due to serotonergic antidepressants

**DOI:** 10.1186/s12991-023-00447-0

**Published:** 2023-04-21

**Authors:** Joseph Ben-Sheetrit, Yehonathan Hermon, Shlomo Birkenfeld, Yehiel Gutman, Antonei B. Csoka, Paz Toren

**Affiliations:** 1 Tel-Aviv Brüll Community Mental Health Center, Clalit Health Services, 9 Hatzvi St., 6719709 Tel-Aviv, Israel; 2 Geha Mental Health Center, Petah Tikva, Israel; 3 Clalit Health Services, Tel-Aviv, Israel; 4 Sackler Faculty of Medicine, Tel-Aviv University, Tel-Aviv, Israel; 5 Department of Anatomy, School of Medicine, Howard University, Washington DC, US

**Keywords:** Selective Serotonin Reuptake Inhibitors (SSRIs), Erectile Dysfunction (ED), Serotonergic Antidepressants, Post-SSRI Sexual Dysfunction (PSSD)

## Abstract

**Background:**

Sexual dysfunction is a common side effect of Serotonergic antidepressants (SA) treatment, and persists in some patients despite drug discontinuation, a condition termed post-SSRI sexual dysfunction (PSSD). The risk for PSSD is unknown but is thought to be rare and difficult to assess. This study aims to estimate the risk of erectile dysfunction (ED) and PSSD in males treated with SAs.

**Methods:**

A 19-year retrospective cohort analysis was conducted using a computerized database of the largest HMO in Israel. ED was defined by phosphodiesterase-5 inhibitors prescriptions. 12,302 males aged 21–49 met the following criteria: non-smokers, no medical or psychiatric comorbidities or medications associated with ED, no alcohol or substance use. Logistic regression was used for estimation of ED risk in SA-treated subjects compared to non-SA-treated controls, assessed with and without the effects of age, body mass index (BMI), socioeconomic status (SES), depression and anxiety, yielding crude and adjusted odds ratios (cOR and aOR, respectively).

**Results:**

SAs were associated with an increased risk for ED (cOR = 3.6, p < 0.000001, 95% CI  2.8–4.8), which remained significant after adjusting for age, SES, BMI, depression and anxiety (aOR = 3.2, p < 0.000001, 95% CI  2.3–4.4). The risk for PSSD was 1 in 216 patients (0.46%) treated with SAs. The prevalence of PSSD was 4.3 per 100,000.

**Conclusions:**

This work offers a first assessment of the small but significant risk of irreversible ED associated with the most commonly prescribed class of antidepressants which should enhance the process of receiving adequate informed consent for therapy.

## Background

Sexual dysfunction is a well-documented side effect of several classes of serotonergic antidepressants (i.e., acting at least partially on serotonin), including the selective serotonin reuptake inhibitors (SSRIs), serotonin norepinephrine reuptake inhibitors (SNRIs), and tricyclic antidepressants (TCAs) [1, 2]. Sexual dysfunction can negatively affect a person’s quality of life, relationships and self-esteem. It may also result in lower satisfaction with antidepressant treatment, leading to medication noncompliance and hindered recovery [1]. Serotonergic antidepressants are used to treat a wide variety of psychiatric disorders, including depressive and anxiety disorders, obsessive–compulsive disorder (OCD), post-traumatic stress disorder (PTSD), and eating disorders, as well as several non-psychiatric disorders, including chronic pain conditions, migraines, sleep disorders, and premature ejaculation [3–6]. Data suggest that more than one in every eight Americans have used an antidepressant, with serotonergic antidepressants the most frequently used [7]. The fact that serotonergic antidepressants have been associated with disturbances in every aspect of sexual function, including libido, arousal, erection, lubrication, orgasm and genital sensation [8], is thus of prime clinical importance. Of the various types of sexual dysfunction that can arise during antidepressant treatment, erectile dysfunction (ED) is perhaps unique in that it has a specific class of pharmacological antidotes indicated for its treatment, namely the phosphodiesterase-5 (PDE-5) inhibitors (i.e., sildenafil, tadalafil and vardenafil). Several studies have documented the association between serotonergic antidepressants and ED with incidences of 10–34% of treated patients [9].

Although sexual dysfunction induced by serotonergic antidepressants is commonly assumed to resolves upon drug discontinuation, it persists in some patients despite cessation of antidepressant treatment, a condition known as post-SSRI sexual dysfunction (PSSD) [5, 8, 10, 12]. It should be noted that although its name would suggest that PSSD refers only to SSRIs, it has been described with other antidepressant that act on serotonin (e.g., serotonin-norepinephrine reuptake inhibitors [8, 13], nephzodone [11]) and is defined here as such. Finally, in June of 2019, PSSD gained an official recognition by the European Medicines Agency as a sexual dysfunction that can endure after SSRI\SNRI treatment stops [14]. Symptoms such as decreased libido, loss of genital sensation (i.e., genital anesthesia) and pleasureless orgasm are characteristic of PSSD [4, 8, 15, 16]. In males, PSSD can also be associated with varying degrees of erectile dysfunction [9]. The duration of PSSD can vary in length; in some prospective randomized trials, SSRI-induced sexual dysfunction persisted for as long as 6 months after drug discontinuation [17], while several case studies and at least two large case series demonstrated that PSSD can persist indefinitely for many years after drug discontinuation in the absence of active depression or anxiety [4, 8, 15]. The duration of use of antidepressants that led to PSSD in a previous study was anywhere between four days to 2.5 years; the duration of PSSD after drug discontinuation ranged from one month to 16 years (at the time of the study) [8].

Animal studies support the pharmacological basis of PSSD. In a systematic review of 14 placebo-controlled trials in rodents concluded that early exposure to SSRIs (e.g., during the neonatal period) was associated with permanent sexual dysfunction persisting after drug discontinuation, including no mounting (RR  0.73, 95% CI  0.62–0.86), no intromission (RR  0.74, 95% CI  0.60–0.92) and no ejaculation behaviors (RR  0.49, 95% CI  0.24–1.00) [18]. Although the exact mechanism of PSSD is unknown, several theories have been suggested [8, 19, 20]. There is currently no established treatment for PSSD, although there are case reports of various improvements with different treatments for specific symptoms [4, 15]. Several studies suggest that PSSD is a severe and irreversible adverse event associated with a pervasive negative impact on the quality of life [4, 8, 15, 16, 20, 21]. Since the recognition of PSSD by the European Medicines Agency in 2019, a significant amount of evidence for PSSD has accumulated. However, no definitive answers about the risk and prevalence of PSSD have been found which makes it difficult to communicate the risk to patients as part of the informed consent process [16, 21, 22].

The objectives of this study were to: (1) to assess the risk of erectile dysfunction (as treatment with PDE-5 inhibitors) in males with and without a history of treatment with serotonergic antidepressants, in the absence of medical or psychiatric comorbidities or other medications associated with ED, and (2) to estimate the prevalence of post-SSRI sexual dysfunction (PSSD) in males, as evidenced by treatment-emergent ED treated with PDE-5 inhibitors persisting after discontinuation of antidepressant treatment and no active diagnosis of depression or anxiety.

## Methods

### Procedure

In this study, we used the computerized database of Clalit Health Services (CHS), Tel-Aviv district, Israel. The validity of the diagnoses in the database was previously found to be high [23, 24]. The study was approved by the CHS Institutional Review Board (IRB). Erectile dysfunction was defined as one or more prescriptions of a PDE-5 inhibitor (i.e., sildenafil, tadalafil, vardenafil) during the study period. Inclusion criteria were: (1) male, (2) 20 < age < 50 years-old, (3) a documented last BMI < 25. To avoid the possibility of undiagnosed age-related medical conditions causing ED, we chose not to include participants over the age of 49. Exclusion criteria were: (1) medical or psychiatric comorbidities associated with ED (other than unipolar depression and anxiety disorders, see below), (2) treatment with medications known to cause ED (other than antidepressants, see below) during the entire study period, (3) past or present use of 5α-reductase inhibitors (i.e., finasteride or dutasteride; in order to avoid potential cases of Post-finasteride syndrome [25]), and (4) history of alcohol or substance use disorders, and (5) current or past smoking.

### Medical and psychiatric exclusion criteria

Medical diagnoses that led to exclusion in our sample were any cardiovascular (e.g., ischemic heart disease, hypertension, congestive heart failure, valvular heart disease, cardiomyopathy, aortic coarctation, peripheral vascular disease), cerebrovascular (e.g., transient ischemic attack, cerebrovascular accident), neurologic (e.g., epilepsy, dementia, cerebral palsy, post-concussion syndrome, polyneuropathy, myasthenia gravis, migraines, cluster headache, spinal cord injury, amyotrophic lateral sclerosis), metabolic and endocrine (e.g., impaired fasting glucose, diabetes mellitus, diabetes insipidus, obesity, hypothyroidism, thyrotoxicosis, Cushing syndrome, Addison’s disease, acromegaly, hypopituitarism, hypogonadism), severe respiratory (e.g., chronic obstructive pulmonary disease, cystic fibrosis, pulmonary hypertension, tuberculosis), rheumatologic (e.g., systemic lupus erythematosus, rheumatoid arthritis, gout, antiphospholipid antibody syndrome, polymyalgia rheumatic, sarcoidosis, Behcet’s syndrome), severe dermatologic (e.g., psoriasis, scleroderma, pemphigus vulgaris), chronic or severe infectious (e.g., human immunodeficiency virus, chronic viral hepatitis, syphilis, gonorrhea), oncologic (e.g. hematologic malignancies, malignant neoplasms, benign neoplasms of genitourinary tract), post-transplantation status, severe orthopedic (e.g., spinal stenosis, limb amputation), gastrointestinal and hepatic (e.g. ulcerative colitis, Crohn’s disease, celiac disease, cirrhosis, hepatic failure, autoimmune hepatitis, rectal or anal surgical disorders) and genitourinary (e.g., chronic kidney disease, prostatic disorders, Peyronie’s disease, genital anomalies) disorders that were either associated with ED in the literature [26] or that could plausibly lead to ED on the basis of clinical considerations. Subjects with a history of schizophrenia, bipolar disorder, autism, intellectual disability, major neurocognitive disorders (dementia), PTSD, OCD, dissociative disorders, personality disorders, somatoform disorders or gender dysphoria were excluded [27]. Any history of dependence or abuse of alcohol, cannabis, other illicit drugs or prescription medications led to exclusion. Subjects with an active diagnosis of depression or anxiety, as per CHS database, were excluded for reasons of possible confounding effects of depression and anxiety on erectile dysfunction. It should be noted that excluding by diagnosis allows to identify further cases i.e., patients who received only psychotherapy, or patients with untreated chronic depression. Subjects who discontinued their prescribed antidepressants, i.e., non-compliance, were excluded in order to avoid cases in which the primary cause of ED might have been a possible exacerbation of depression and anxiety.

### Medications leading to exclusion

Subjects who have taken any of the following medications [26] were excluded: angiotensin converting enzyme (ACE) inhibitors, alpha blockers, beta blockers, calcium channel blockers (CCB), angiotensin-II receptor blockers (ARBs), vasodilators used in cardiac diseases (e.g. isosorbide dinitrate), diuretics (not including furosemide [28]), antiarrhythmic medications (class I and III), digoxin, hormones and hormone analogs, hormone antagonists and related agents, statins, fibrates, antineoplastic agents, immunosuppressants, immunostimulants, anticonvulsive and mood-stabilizing agents (not including topiramate [29] and lamotrigine [30]), antipsychotics, anticholinergic drugs used for urinary conditions, systemic steroid (besides cases of a single prescription), opioids (besides cases with a single prescription), amphetamines, and drugs used in addictive disorders.

In order to prevent misclassification of chronic pre-treatment erectile dysfunction as PSSD, subjects who had been prescribed PDE-5 inhibitors before they were prescribed antidepressants were excluded.

### Measures

#### Demographic variables

Age and BMI were retrieved from the medical database. We defined a 3-level Socioeconomic status (SES) variable based on exemption from Social Security Tax (SST) and billing approval by the CHS. Exemption from payment of the SST in Israel is usually based on having a low income or documented disability, or otherwise belonging to specific populations of lower SES (e.g. new immigrants), and is considered a strong indication of a low SES. Additionally, some of the CHS members have an authorization to pay for healthcare services by means of direct billing of their bank account, which is usually an indication of higher SES. Based on these data, each patient was assigned to one of the following groups: High (no SST exemption, direct billing approved), Middle (no SST exemption, direct billing not approved) or Low SES (exemption of SST payment, regardless of billing approval). It should be noted that a “High SES” does not necessarily mean a high SES compared to the general Israeli population.

#### Post-SSRI sexual dysfunction

Based on the previous literature [8, 14], we defined PSSD as treatment-emergent sexual dysfunction following the use of one or more serotonergic antidepressants that persists despite the cessation of antidepressant treatment for one month or more. In identification of cases of PSSD, it is important to address possible confounders such as chronic pre-treatment sexual dysfunction, common causes of sexual dysfunction unrelated to antidepressants, and the possible persistence of sexual dysfunction due to active non-treated major depression. To overcome this hurdle, we excluded subjects with a wide array of medical and psychiatric disorders or medications that may be associated with ED (see above). It should be noted that many of these diagnoses and medications may not pose a contra-indication for the diagnosis of PSSD in clinical practice, where the clinical course and symptom profile of the particular patient are available. According to a previous study [31], the mean delay in seeking medication for ED is around 30 months from the onset of ED. However, we defined a maximum of 12 months from the first use of antidepressants to the first use of PDE-5 inhibitors as an indicator of treatment-related erectile dysfunction, thus minimizing the risk of misidentifying PDE-5 inhibitor prescriptions unrelated to antidepressants as indicating PSSD.

### Statistical analysis

We used SPSS ver. 22 (Chicago, IL, USA) for statistical analysis.

Prior to the main analysis, preliminary analyses were conducted to compare the study and control groups on key demographic and clinical characteristics. Independent samples t-tests were used to compare the groups on age and BMI, while chi-square tests were used to compare the groups on socioeconomic status (SES). Chi-square tests for independence were also used to evaluate between-group differences in the use of PDE-5 inhibitors.

The main analysis involved two steps. First, chi-square tests for independence were used to compare the study and control groups on their use of PDE-5 inhibitors. Second, a logistic regression model was fitted to assess the association between history of serotonergic antidepressant use during the study period and the risk of developing erectile dysfunction, as indicated by a prescription for a PDE-5 inhibitor. Two sets of logistic regression models were estimated, one using crude odds ratios (cOR) and the other using adjusted odds ratios (aOR) that controlled for potential confounding variables including age, SES, BMI, depression, and anxiety disorders.

## Results

### Sample characteristics

**Fig. 1:**
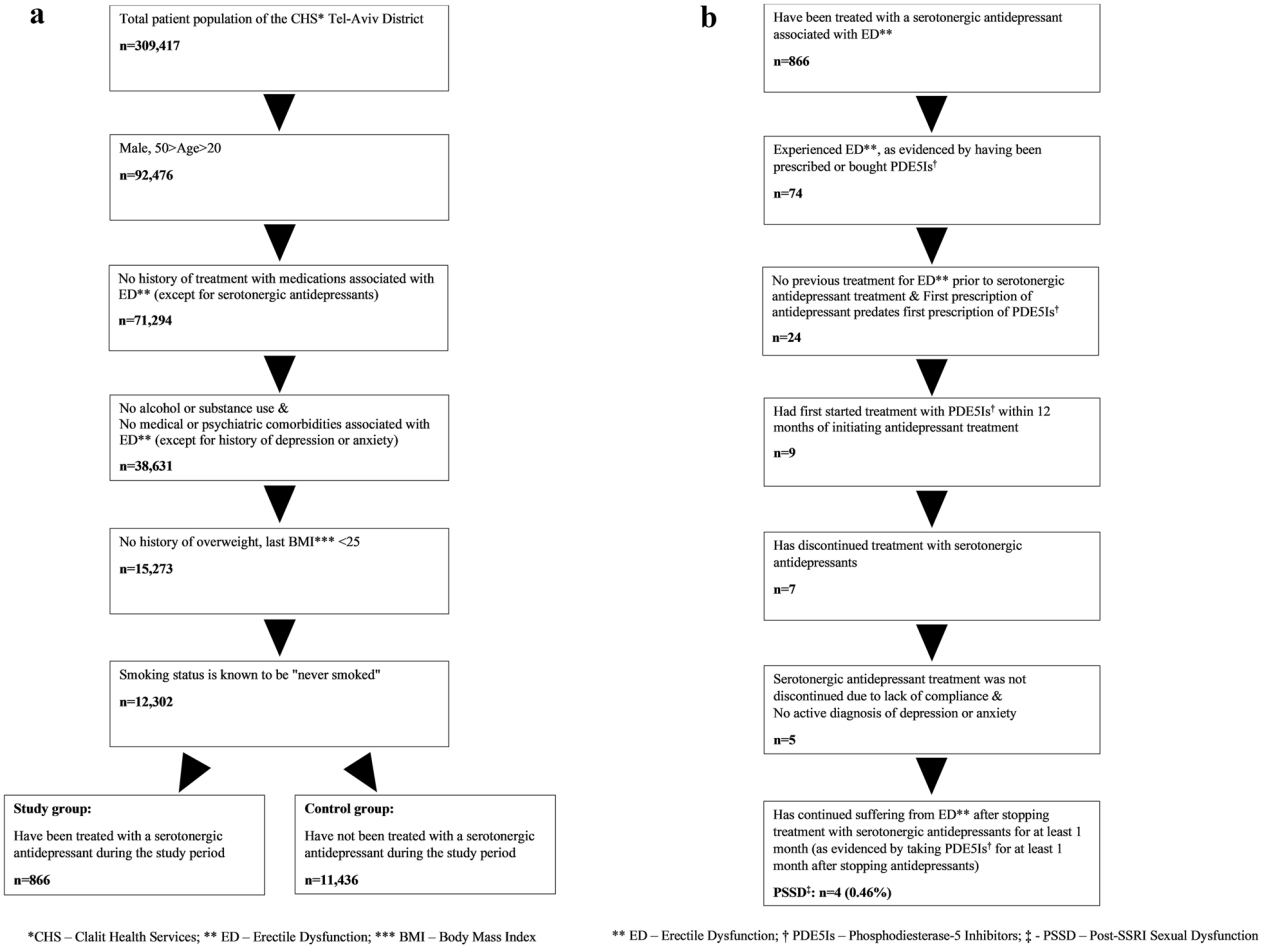
**A** Subject flow chart: From population to Study & Control groups. **B** Study subject flow chart

Inclusion and exclusion criteria were met by 12,302 males, of whom 866 (7.0%) had used serotonergic antidepressants during the study period (i.e., the study group) and 11,436 had not (i.e., the control group). For the subject flow chart from population to Study & Control groups see Fig. 1A. The mean age was 34.5 ± 6.5 years in the study group, and 33.4 ± 6.2 years in the control group (t_(12,300)_ = −5.0, p < 0.001). The proportion of subjects assigned to each SES category differed significantly between the study and control groups [χ^2^(2) = 31.6, p < 0.001), with the study group having a higher proportion of participants in the High SES category (67.7%) compared to the control group (65.1%). The number of participants and percentage in each SES category are shown separately in Table 1. The mean BMI was slightly but significantly lower in the study group [M = 22.0 (2.0)] compared to the control group [M = 22.2 (1.9); t(12,300) = 2.8, p < 0.05].

**Table 1:** Sample characteristics

	Study group (n = 866)	Control group (n = 11,436)	Group differences
Demographic variables			
Age (years)—M (SD)	34.5 (6.5)	33.4 (6.2)	t_(12,300)_ = -5.0, p < 0.001
SES Category—n (%)			χ^2^_(2)_ = 31.6, p < 0.001
High	586 (67.7%)	7446 (65.1%)	
Middle	253 (29.2%)	3867 (33.8%)	
Low	27 (3.1%)	123 (1.1%)	
BMI—M (SD)	22.0 (2.0)	22.2 (1.9)	t_(12,300)_ = 2.8, p < 0.05

Values are presented as mean (SD) or n (%)

*SES* socioeconomic status, *BMI* body mass index

### Use of antidepressant medications in the study group

The number of antidepressants used per patient in the study group during the study period is reported in Table 2. The majority of patients (76.4%) used only one antidepressant during the study period. The types of serotonergic antidepressants used during the study period varied, with SSRIs being the most frequently used (79.9%).

**Table 2:** Breakdown of antidepressants use in the study group (n = 866)

Antidepressants used per patient—n (%)	
One	662 (76.4%)
Two	148 (17.1%)
Three	48 (5.5%)
Four or More	26 (0.9%)

Serotonergic antidepressants used—n (%)	
SSRIs	892 (79.9%)
TCAs	135 (15.6%)
SNRIs	70 (8.1%)
Hypericum	53 (6.1%)
Mirtazapine	23 (2.7%)
Mianserin	13 (1.5%)
Trazodone	7 (0.8%)
Vortioxetine	3 (0.3%)
Maprotiline	2 (0.2%)
Phenelzine	1 (0.1%)

### Group differences in the use of PDE-5 inhibitors

Table 3 presents the study characteristics related to PDE-5 inhibitor use during the study period. Of the total 12,302 study sample subjects, 360 subjects (2.9%) had used a PDE-5 inhibitor during the study period, including 74 subjects (8.5%) in the study group and 286 (2.5%) controls (χ^2^_(1)_ = 103.5, p < 0.001). One hundred and eighty seven subjects (51.9%) used one type of PDE-5 inhibitor, whereas 115 (31.9%) used two and 58 (16.1%) used all three PDE-5 inhibitors (sildenafil, tadalafil and vardenafil) during the study period. The most commonly used PDE-5 inhibitor was sildenafil (n = 245, 68.1%), followed by an equal number of users of tadalafil and vardenafil (n = 173, 48.1% each).

**Table 3:** Comparison of PDE-5 use between groups

	Study Group (n = 866)	Control Group (n = 11,436)	Total (n = 12,302)	Group differences
PDE-5 Inhibitor Use	74 (8.5%)	286 (2.5%)	360 (2.9%)	χ^2^_(1)_ = 103.5, *p* < 0.001
Number of PDE-5 Inhibitors Used				
One			187 (51.9%)	
Two			115 (31.9%)	
Three			58 (16.1%)	
Types of PDE-5 Inhibitors Used—n (%)				
Sildenafil			245 (68.1%)	
Tadalafil			173 (48.1%)	
Vardenafil			173 (48.1%)	

Values are presented as n (%)

*PDE-5* phosphodiesterase type 5

### Prediction of PDE-5 inhibitor use

The results of the logistic regression are reported in Table 4. Serotonergic antidepressant use was significantly associated with PDE-5 inhibitor use (OR = 3.6 [2.8, 4.8], p < 0.001), and this association remained significant after adjusting for age, SES, BMI, depression, and anxiety (OR = 3.2 [2.3, 4.4], p < 0.001). Age and BMI also had small but significant effects on ED risk (OR = 1.08 [1.06, 1.1], p < 0.001 and OR = 1.07 [1.01, 1.1], p = 0.02, respectively). Compared to subjects of high SES, subjects of middle SES had a lower risk for ED (OR = 0.7 [0.6, 0.9], p = 0.01), but there was no statistically significant difference compared to subjects of low SES. Depression and anxiety had no significant effect on the risk of ED beyond the effects of antidepressant use, age, BMI, and SES. The model including only the Serotonergic Antidepressant Use was significant (χ^2^_(1)_ = 71.8, p < 0.001), Nagelkerke R^2^ was 0.03, and the model correctly classified 97.1% of cases.

**Table 4:** Logistic regression analysis of factors associated with PDE-5 inhibitor use

Variable	Crude Odds Ratio (cOR) [95%CI]	p-value	Adjusted Odds Ratio (aOR) [95%CI]	p-value
Serotonergic Antidepressant Use	3.6 [2.8, 4.8]	< 0.001	3.2 [2.3, 4.4]	< 0.001
Age	–		1.08 [1.06, 1.1]	< 0.001
SES	–			
High (reference)	–			
Middle	–		0.7 [0.6, 0.9]	0.01
Low	–			
BMI	–		1.07 [1.01, 1.1]	0.02

*OR* odds ratio, *CI* confidence interval, *SES* socioeconomic status.

### Post-SSRI Sexual Dysfunction

Out of the 866 subjects of the study group, four subjects (0.46%) met full case criteria for PSSD (see Fig. 1B). These subjects were 32, 34, 37, and 42 years-old. They used antidepressants with at least a partial serotonergic mechanism (escitalopram, paroxetine, imipramine, or nortriptyline) for 4–8 months before initiating treatment for erectile dysfunction with PDE-5 inhibitors (all used tadalafil and vardenafil, but not sildenafil). They continued to have erectile dysfunction after drug discontinuation as evidenced by 3.6–14.8 months of PDE-5 inhibitor treatment at the end of the study period. The prevalence of PSSD in males of ages 21–49 years of the population of the CHS Tel-Aviv district is 4.3 per 100,000 (4 in 92,476).

## Discussion

In this study, we found that after exclusion of confounding factors, treatment with serotonergic antidepressants was associated with more than a threefold increase in the risk of receiving PDE-5-inhibitor treatment for ED compared to controls, and that PSSD developed in 0.46% (1 in every 216) of the patients who were treated with SAs. The prevalence of PSSD in the CHS Tel-Aviv district population of males aged 21–49 years according to our study is 4.3 per 100,000. The findings support the previous literature on PSSD, and indicate that treatment with serotonergic antidepressants is associated with a small but significant risk of irreversible sexual dysfunction after drug discontinuation.

### Erectile dysfunction

The fact that the association between serotonergic antidepressants and ED remained robust even after rigorous exclusion of confounding factors adds to the existing literature and highlights the importance of detecting and addressing antidepressant-induced ED. The very small effects of age and BMI on ED in our study may be explained by the initial strict inclusion of young males with normal BMIs. Our finding of decreased risk of ED in males of middle compared to high SES differs from those of previous studies showing lower rates of ED in males of high SES [32]. This may be explained by the more rigorous exclusion of medical and psychiatric comorbidities in our study, as the relationship between high SES and lower risk for ED has been hypothesized to be due to overall better health and less medical morbidity in persons of high SES. Our findings suggest that when medical morbidity is thoroughly taken into account, higher SES per se does not confer a defense against ED, and may in fact be associated with somewhat increased risk for ED, reminiscent of the increased risk for premature ejaculation in subjects of high SES (college-educated subjects) [33]. It could be that the higher risk of PDE-5 inhibitor treatment in patients with high SES may perhaps be due to higher awareness and referral for treatment.

### Post-SSRI sexual dysfunction

The issue of antidepressant-induced sexual dysfunction in general and PSSD in particular illustrates the limitations of the paradigm of short-term clinical trials with regard to drug safety. Based on randomized clinical trials relying on self-report, sexual dysfunction was first considered an uncommon side effect of antidepressants, only to be revealed in later studies as the most common long-term adverse effect of the SSRIs when evaluated using specific questioning [34]. In addition to being a highly sensitive topic, persistent sexual dysfunction with SSRIs may have escaped clinical notice until the early 2000s because of numerous factors. PSSD is sufficiently rare to make most if not all clinical trials under-powered to discover it. PSSD is only clearly evident after a significant amount of time has passed from treatment onset, including time for sexual dysfunction detection, drug discontinuation, and a wait-and-see period for possible recovery (which fails)—making most studies too short-termed to capture it. Moreover, unlike other forms of drug-induced persistent sexual dysfunction, such as the post-finasteride syndrome (PFS), most (but not all) antidepressant prescriptions are for treatment of depressive or anxiety disorders, which can be associated with sexual dysfunction themselves, thus making it difficult to realize that the drug-induced basis of this phenomenon. Finally, the common wisdom that drug effects can only manifest when the drugs are actively circulating within the body has probably hampered the ability of clinicians to consider PSSD for many years. This belief is contradicted by several potentially irreversible drug-induced adverse effects, such as tardive dyskinesia, amiodarone lung and post-finasteride syndrome [25], that can persist for years after drug discontinuation. Taken together, the PSSD syndrome emphasizes the need for structured, long-term post-marketing surveillance of all drugs in order to be able to capture a fuller picture of their effects, both therapeutic and otherwise.

### Limitations

The current study is limited by its retrospective design; its relatively-narrow focus on men only; its focus on erectile dysfunction rather than other forms of sexual dysfunction; and the relative reliance on physician-diagnosed conditions for medical history. However, it is inherently difficult to detect a rare and long-term phenomenon such as PSSD in prospective studies. The focus on ED was necessary in order to be able to detect sexual dysfunction beyond self-report, but we acknowledge that it may underestimate the true prevalence of PSSD. Further, our estimation may reflect the risk in otherwise healthy individuals and may again represent an underestimation of the prevalence of PSSD in the general population. Furthermore, the reliance on PDE-5 inhibitor treatment as a measure of ED sets a high threshold for detection and is probably also an underestimation of antidepressant-induced ED, as many patients may not seek medical help due to shame or unawareness. Finally, it should be noted that the double-exclusion of patients with comorbidities by diagnosis *or* associated medications (e.g., by excluding all patients who have hypothyroidism *or* who have ever used levothyroxine) minimizes the limitation of reliance on physician-diagnosed conditions.

## Conclusion

In conclusion, our findings indicate that serotonergic antidepressants carry a small but significant risk of about 0.46% of developing an irreversible sexual dysfunction persisting after their discontinuation (post-SSRI sexual dysfunction, PSSD). As a long-term sexual disability, PSSD is a serious adverse effect of treatment with serotonergic antidepressants, and patients should be informed of its risk before their prescription. Our findings allow a clearer communication of the risk of PSSD to patients who are considering treatment with SSRIs, SNRIs and other serotonergic antidepressants. The findings highlight the importance of measuring sexual function not only before and during antidepressant treatment, but also after treatment discontinuation, in order to be able to detect PSSD. However, the diagnosis of PSSD should be made only after thoroughly considering the timeline and course of clinical manifestations and performing a comprehensive workup [15], including complete physical examination, appropriate laboratory tests (e.g., fasting glucose or HbA1c, blood hormone levels, imaging studies when indicated), and psychiatric evaluation. Moreover, PSSD should not be diagnosed before appropriate time for recovery has elapsed after drug discontinuation (e.g., one month) [8]. A new-onset genital anesthesia [8, 35], and sexual dysfunction that emerges or worsens despite a clear improvement in depression and anxiety during drug therapy [8], may support the diagnosis of PSSD, although they are neither necessary nor sufficient for diagnosis. For reviews of PSSD including anecdotal evidence for possible treatments in documented cases, see Reisman (2017) [4], and Bala et al. (2017) [15]. Currently, the only established treatment for PSSD is prevention. Because genital anesthesia is common in PSSD [4, 8, 15], some experts have recommended that treatment with SSRIs should be discontinued as soon as genital anesthesia develops [35].

The main conclusion from this study is the importance of openly discussing the risk of PSSD and alternative treatments to serotonergic antidepressants with at risk patients as part of the informed consent process.

## Data Availability

The datasets generated and/or analyzed during the current study are not publicly available due to CHS institutional review board confidentiality regulations.

## Abbreviations

ACE: Angiotensin Converting Enzyme
BMI: Body Mass Index
CCB: Calcium Channel Blockers
CHS: Clalit Health Services
ED: Erectile Dysfunction
IRB: Institutional Review Board
OCD: Obsessive–Compulsive Disorder
PDE-5: Phosphodiesterase-5
PFS: Post-Finasteride Dyndrome
PSSD: Post-SSRI Sexual Dysfunction
PTSD: Post-Traumatic Stress Disorder
SA: Serotonergic Antidepressants
SES: Socioeconomic Status
SNRI: Serotonin Norepinephrine Reuptake Inhibitor
SSRI: Selective Serotonin Reuptake Inhibitor
SST: Social Security Tax
TCA: Tricyclic Antidepressant
